# Supplementary material for: Are carbon emissions trading and green financial instruments synergistic? -Comprehensive quantitative research based on content analysis
Source: PLoS One. 2024 Mar 7;19(3):e0298601. doi: 10.1371/journal.pone.0298601 (PMC10919601; doi:10.1371/journal.pone.0298601)
Supplement: S1 Data — (ZIP) [file pone.0298601.s001.zip › Data/地级市Z_F工作报告.zip!/╔╜╢1⁄2/╘μ╫»╩╨/└┤╫╘╣1⁄2╓┌║┼í╛┬φ┐╦╩2╛▌═°í┐_2015.docx]

各位代表：

现在，我代表市人民政府向大会报告工作，请予审议，并请市政协各位委员提出意见。

一、2014年政府工作回顾

过去的一年，是宏观经济形势错综复杂、经济下行压力持续加大的一年。面对前所未有的困难和挑战，全市上下在市委的正确领导下，千方百计稳增长、促改革、调结构、惠民生，经济社会发展缓中趋稳、稳中有进，转型升级和经济文化融合发展取得了新的成就。全市生产总值完成1980亿元，增长9%；公共财政预算收入完成137.9亿元，增长5.5%；固定资产投资完成1430亿元，增长15.5%；社会消费品零售总额达到706.3亿元，增长12.7%；城镇居民人均可支配收入27596元，增长9.3%；农民人均纯收入12145元，增长11.6%，连续五年高于城镇居民收入增幅。

重点改革稳步推进。围绕打赢市属工业企业三年改革攻坚战，坚持破产改制、资产盘活、产业培育一体推进，连续打出改革组合拳，70家企业改革主体任务基本完成，近4万名困难职工的历史遗留问题基本得到解决；市南工业区建设发展步伐加快，一批文化产业和制造业项目陆续落地，正在成长为转型升级的新引擎。持续推进简政放权，出台了投资项目负面清单、行政审批事项目录清单和行政权力清单，激发了市场活力。全年新登记市场主体3.4万户、注册资本248亿元，分别增长19%和73%。不断深化财税体制改革，营改增试点平稳扩面，煤炭资源税从价计征启动实施；专项转移支付项目压减43%，预算绩效管理得到加强；市级过桥还贷资金累计滚动拨付8.9亿元。加快金融改革步伐，有序开展小额贷款保证保险试点，青岛银行枣庄分行、枣庄农商银行挂牌营业，日照银行枣庄分行正在筹建。扎实推进国家农村改革试验区建设，农村土地承包经营权确权登记完成95.2%；土地合作社数量、土地流转面积、农地抵押贷款数额分别增长46%、25%和26%；市、区(市)、镇(街)三级农村产权交易服务体系基本建成。

结构调整步伐加快。五管齐下推进工业转型振兴，工业经济运行质效稳步提升。全市规模以上工业增加值增长10.5%，制造业增加值占比达到78.7%；纳税前50强企业中，制造业企业纳税额占比达到46%。水泥行业市场化重组效果明显，在全国水泥行业整体不景气的情况下，参与重组企业的利润、税金分别增长110%和65%；中联水泥集团运营中心投入运营。促成山能集团并购重组丰源轮胎，产能达到1000万条。联想化工(一期)、薛城能源主体项目竣工投产，煤化工产业对经济的支撑作用明显增强。锂电、医药、装备制造等新兴产业发展势头良好，高新技术产业产值比重提高到18.8%。工业技改完成投资310亿元，增长15.8%。市产业技术协同创新中心挂牌运行，省级工程技术研究中心达到16家、企业技术中心达到43家、工程实验室达到5家。创新扶持实体经济发展的方式，设立了政府股权投资引导基金，建成启用中小企业公共服务平台。全域化布局乡村旅游，“一园三镇五点”的发展格局初步形成，全市旅游综合收入增长14.1%。微山湖古镇(一期)、红星美凯龙家居广场等一批服务业重点项目建成运营，真爱商城、万洲浙商城等一批项目顺利推进。新发展家庭农场96家、规模以上农业龙头企业36家，新认证“三品一标”农产品32个，我市整体创建成为国家现代农业示范区。

城乡建设协调并进。统筹城乡规划管理，编制全市新型城镇化规划、中心城区整体风貌规划、城市绿地系统规划，城乡建设更加有序。统筹中心城区功能设施建设，2012年以来累计完成城市基础设施建设投入64.3亿元；新城实验小学、市实验高中、市职业中专、市中医医院新院、市立医院新院、羊庄水源地供水工程建成启用，文体中心、市妇保院新院、市社会福利(养老)服务中心、枣矿城等项目顺利推进，教育培训、医疗卫生、文化体育、商贸流通等体系基本形成，城市功能更加完善。统筹城区地上地下设施建设，2012年以来全市新增供热面积1186万平方米，新建改造城区道路196条，整治背街小巷200多条，居民住行更加舒适。统筹小城镇和美丽乡村建设，2个新型城镇化示范镇进入全省考核前20名，12个镇纳入国家级重点镇；70个村达到美丽乡村创建标准；城乡环卫一体化覆盖全市所有村居，城乡环境更加整洁 侯卫东官场笔记。统筹城乡交通建设，客运换乘中心、北留线改建、枣曹线改建等工程进展顺利，通用机场、枣鱼高速跨湖工程纳入全省综合交通网中长期规划，新建改造农村公路373公里，城乡路网更加通畅。

招商引资成效明显。积极开展香港山东周、台湾山东周和面向全国工商联执委的招商活动，鲁南铁路物流中心、穆拉德石榴种植与深加工等一批大项目签约落地，新建外来固定资产投资亿元以上项目143个；百个重点项目完成投资395亿元，泰和水处理等38个项目建成投产，十电扩建、华润三九(二期)等项目进展顺利。枣庄高新区成功创建为国家级高新技术产业开发区，结束了我市没有国家级经济园区的历史。外贸进出口逆势上扬，进出口总额增长15.1%，其中出口增长21.9%、增幅居全省第六位。

生态建设亮点纷呈。自2012年创森工作启动以来，全市共实施重点绿化项目584个、建设中心城区环城绿道200多公里，新增造林绿化面积63.5万亩，成功创建为国家森林城市。扎实推进国家园林城市创建，实施重点园林绿化工程119项。全力打好水污染防治攻坚战，代表山东省参加迎淮核查，取得了淮河流域和全国九大流域“双第一”，受到省政府通报表扬。铁腕治理大气污染，狠抓减煤、降尘、治企、控车等关键环节，改造建成区燃煤锅炉305台，淘汰黄标车、老旧车3.6万辆，全面完成100项工业企业脱硫脱硝治理任务，秸秆焚烧得到有效控制，治超治限工作连续三年居全省前列。围绕让资源环境休养生息，“十二五”以来累计关闭小煤矿、小石膏矿和采石场246处。单位生产总值能耗、主要污染物排放总量继续下降。

民生福祉持续增进。百件惠民实事得到较好落实，全市民生支出120.5亿元，占公共财政支出的比重达到55.5%。新增城镇就业5.2万人，农村劳动力转移就业6.3万人。企业退休人员养老金实现“十连增”。居民基本医疗保险、基本养老保险完成城乡并轨。城市和农村低保标准分别提高到每人每月410元、每人每年2800元，农村五保集中和分散供养标准分别提高到每人每年4500元、3300元。新建社区老年人日间照料中心90处、农村幸福院78处。市中区、峄城区通过国家义务教育发展基本均衡县验收。新建改建标准化校舍26万平方米、幼儿园21处。农村小学、初中生均公用经费标准分别提高到710元和910元。扎实推进县级公立医院综合改革，在全省率先实施公共卫生“健康直通车”服务活动。全民健身工程覆盖2152个村居，人均健身场地达到2.8平方米。新建保障性安居工程9343套，改造农村危房2107户，解决了12.4万农民的饮水安全问题。顺利通过省级文明城市评审，创建成为全省首批“四德”工程示范市。人口自然增长率控制在7.5‰以内。“平安枣庄”建设深入推进，信访工作扎实有效，食品药品监管体系逐步完善；安全生产形势持续稳定，事故起数、死亡人数实现“双下降”。

作风建设得到加强。扎实开展党的群众路线教育实践活动，市政府党组如期完成44项整改任务、30项专项整治内容，达到了干部受教育、行政提效能、群众得实惠的目的。严格执行中央“八项规定”，全市清理违规公务用车675辆次、办公用房4.6万平方米，市级“三公”经费、会议费分别下降13.7%和34%。对2002年以来457件政府规范性文件进行了全面清理。市长热线、市长信箱共受理群众来电来信3.1万件，办结率97%。持续开展“庸懒散”专项整治、“百千百”民主评议和群众满意度调查，从严查处“三乱”行为，营商环境得到优化。完成新一轮政府机构改革，建立了政府法律顾问制度。自觉接受人大、政协监督，102件人大代表建议和538件政协提案全部按时办结。

各位代表，面对近年来全球经济复苏缓慢、国内经济持续下行带来的严峻挑战，面对枣庄发展既要“转”又要“赶”的诸多压力，本届政府始终秉持“打基础、利长远”“民之所望、政之所向”“功成不必在我”的执政理念，胸怀重振老工业基地昔日辉煌的梦想，义无反顾地推进工业转型升级，为枣庄工业的振兴夯实了基础；肩负为子孙后代留下更多青山绿水的使命，下定决心创建国家森林城市和国家园林城市，集中力量治水、治气、治脏，改变了昔日“一黑一灰”的煤城形象；本着对历史负责的态度，统筹推进新老城区建设、地上地下基础设施配套，为城市长远发展提供了支撑；心存让老百姓过上幸福生活的夙愿，千方百计保障和改善民生，使更多的发展成果惠及了百姓。实践证明，我们经受住了复杂形势的考验，拓展了转型发展的空间，取得了来之不易的成绩。这是省委、省政府和市委正确领导的结果，是人大、政协和社会各界监督支持的结果，是全市人民团结一致、顽强拼搏的结果。在此，我代表市人民政府，向奋斗在各行各业的广大干部群众，向解放军、武警驻枣官兵和中央、省驻枣单位，向在我市创业发展的投资者、建设者，向所有关心、支持枣庄发展的港澳台同胞和海内外朋友，表示衷心的感谢和崇高的敬意!

同时，也要清醒地看到，我市发展中还存在一些矛盾和问题：无论是产业还是企业，结构老、新兴少、块头小、链条短、长不大、走不远的问题依然突出。工业经济不强，服务业发展不快，招商引资的氛围不浓，抓项目建设的劲头不足。城市规划建设管理亟需加强，中心城区功能布局分散，小城镇对农村发展的辐射带动乏力，城乡一体化发展任重道远。大气污染防治形势不容乐观，巩固生态建设成果需要付出长期努力。教育、社保、医疗等基本公共服务供给不够均等，许多民生问题还没有得到有效解决。作风建设成果还不稳固，营商环境有待进一步优化，改革创新力度仍需加大，等等。对于这些问题，我们将勇于面对，综合施策，精准发力，切实加以解决，决不辜负全市人民的厚望和重托。二、2015年政府工作总体要求和主要目标

今年政府工作的总体要求是：深入贯彻落实党的十八大、十八届三中四中全会和中央、全省经济工作会议精神，主动适应经济发展新常态，以稳中求进为总基调，以提高经济发展质量和效益为中心，以转方式调结构为主线，以改革开放创新为动力，突出招商引资和项目建设，突出工业提质、农业提效、服务业提速，突出城乡一体同步发展、生态民生同步改善，努力开创转型升级和经济文化融合发展新局面。

今年经济社会发展的主要预期目标是：全市生产总值增长8.5%左右；公共财政预算收入增长8%左右；固定资产投资增长15%左右；社会消费品零售总额增长12%左右；进出口总额增长8%左右；城镇居民人均可支配收入、农民人均纯收入分别增长9%和10%左右；居民消费价格涨幅控制在3%左右；城镇登记失业率控制在3.5%以内；人口自然增长率控制在9.5‰以内；全面完成省下达的年度节能减排约束性目标。

做好今年的工作，需要统筹把握以下五个方面：一是必须主动适应发展新常态。既要深刻理解经济发展新常态的内涵和规律，正确处理速度与质效、现实与预期的关系；更要立足枣庄市情，坚持以好为先、好中求快、又好又快，心无旁骛抓发展，卧薪尝胆图赶超。二是必须探索转型发展新路径。把做大做强主导产业作为抢占转型升级高地的“光明大道”，大力发展实体经济，力促传统产业重塑辉煌、新兴产业绽放光彩、经济文化充分融合，加快由外延式增长向内涵式增长转变、由政府推动型发展向市场决定型发展转变、由过度依赖资源环境向建设资源节约环境友好型社会转变。三是必须激发经济增长新动力。坚持问题导向补短板、变革机制添活力，更加注重运用改革的办法化解矛盾、破解难题，更加注重运用开放的思维借船出海、借力发展，更加注重运用创新的方式驱动产业升级、人才聚集，为加快转型发展、科学发展、赶超发展提供持久动力。四是必须增创区域竞争新优势。牢固树立“营商环境就是生产力、抓环境就是抓发展”的理念，整体推进国家文明城市、国家园林城市、国家卫生城市、国家智慧城市、国家环保模范城市、省级生态市“六城同创”，倾力打造务实高效、清正廉洁的政务环境，公平诚信、监管有力的市场环境，青山绿树、碧水蓝天的生态环境，把品质卓越的“软环境”转化为区域竞争的“硬实力”。五是必须锤炼担当实干新作风。引导各级干部切实增强“为官一任、造福一方”的责任意识，少做观察员和评论员，多做运动员；不当围观的“看客”和发展的“过客”，甘当奉献的“挑夫”和服务的“更夫”，真正动起来、干起来，事事有担当，人人有作为，用实实在在的发展业绩向全市人民交上一份满意的答卷!

三、深入推进各项改革

紧跟中央和省改革步伐，以“敢啃硬骨头”“勇涉深水区”的精神，狠抓各项改革措施的落实。

深化企业改革。继续抓好市属工业企业改革改制扫尾，全面完成既定目标任务。加快推进市南工业区建设发展，集中精力抓好产业招商，打造活力新区。大力发展混合所有制经济，坚持因企施策，舍得拿出“肥肉”，吸引社会资本参与国有企业改革重组，推进国有企业产权多元化。对一般竞争性企业，坚持市场化改革方向，实现国有资本、集体资本、非公有资本交叉持股、相互融合。对公益性企业，以国有控股为主，把社会效益放在首位，着重监管公共产品、公共服务的质量和成本。支持企业经营管理者、技术人员和业务骨干，通过技术入股、增量持股、期股期权等途径入股，构建资本所有者和劳动者利益共同体 完美世界小说。鼓励民营资本通过出资入股、收购股权、认购可转债等方式，参与国企投资项目。健全以管资本为主的国有资产监管模式，实现市属经营性国有资产的统一监管、保值增值。引导企业完善法人治理结构，逐步推行职业经理人管理模式，加快建立现代企业制度。

深化农村改革。加快推进国家农村改革试验区建设，进一步释放“三农”发展活力。以开展土地承包经营权流转管理试点为抓手，完善农村产权交易市场体系，规范土地流转和合作社运行。以拓展农村改革领域为导向，深化集体产权股份制改革，全面完成土地承包经营权、集体经营性建设用地和宅基地使用权确权登记颁证任务，赋予农民更多财产权利。以保障农业发展为目标，进一步搞好农民信用合作，开展马铃薯、生猪目标价格保险试点，为农业生产提供“助力器”和“保障网”；稳步推进供销社综合改革，抓好村级供销服务中心建设，促进“三农”服务便利化。

深化行政审批改革。严格落实投资项目负面清单、行政审批事项目录清单和行政权力清单，出台政府部门责任清单，完善政务服务平台，构建“四清单一平台”行政权力运行体系。承接好中央和省下放的行政审批事项，再取消下放一批市级行政审批事项。继续清理规范行政事业性收费、经营服务性收费和政府性基金项目，在现有基础上再压减三分之一以上。深化商事制度改革，逐步推行营业执照、组织机构代码证、税务登记证“三证合一”，培育发展各类市场主体。加快社会征信体系建设，实施统一社会信用代码制度，让诚实守信者得到保护、作假失信者受到惩戒。

深化财税体制改革。今年起，市、区(市)两级全面公开政府预决算，所有部门都要公开部门预决算和“三公”经费预决算。改进和创新财政支持经济社会发展方式，采取基金引导、购买服务、PPP等多种方式，提高财政资金使用效率。管好用好政府股权投资引导基金，争取三年内规模达到10亿元以上。加强财税征收管理，合理统筹财政收入增速、税收占比和“四税”占比，提高财政收入质量。调整优化支出结构，大力压减投向一般竞争性领域的资金，集中财力保民生、保改革、保重点项目、保法定支出。加强政府性债务管理，确保不发生区域性风险。

深化金融改革。加大异地金融机构特别是股份制商业银行引进力度。支持地方银行引入战略投资伙伴，推动增资扩股、跨区域经营。大力发展普惠金融，推动民间资本管理公司、民间资本服务中心县域全覆盖。鼓励传统金融机构与互联网企业、电子商务企业开展深度合作、跨界发展。加大对企业上市的辅导力度，引导符合条件的企业到场外交易市场挂牌融资。支持企业发行短期融资券、中期票据、集合票据和境外融资，开展好中小企业私募债发行试点工作，提高金融服务实体经济水平。整顿和规范金融市场秩序，依法打击金融违法犯罪行为。

深化其他领域改革。加快事业单位分类改革，基本完成经营类事业单位转企改制，推进行政类事业单位改革。探索事业单位管办分离，规范中介机构管理，加快行业协会、商会与行政机关脱钩。将社会力量创办的非营利性学校、医院、养老等机构，逐步纳入事业单位法人登记管理。推进机关事业单位工资和养老保险制度改革，实行县以下职务职级并行制度，提高基层公务人员工作补贴。全面完成市、区(市)两级党政机关公务用车制度改革。稳妥推进价格改革，完善主要由市场决定价格的机制。

四、加快产业转型升级

实现枣庄的转型发展，产业转型是关键，企业转型是基础。在全面摸清我市产业现状的基础上，深入实施行业、企业对标挖潜，推动传统产业做优、新兴产业做大、龙头企业做强、关联企业做精，加速培育千亿级、百亿级的产业集群和企业集团，打造枣庄实体经济发展的新舰队。

加快改造提升传统产业。继续大力实施工业转型振兴计划，通过整合重组、技术改造等方式，让“老产业”迸发新活力。煤化工产业，依托鲁南煤化工、薛城能源等产业园区，加快瑞达聚丁烯、奥瑟亚建阳炭黑等项目建设，把产品往下游发展、把链条往高端延伸，抢占煤气化、煤焦化两个领域的制高点，打造国内重要的煤基新材料基地。水泥产业，巩固好市场化重组成果，扩大重组覆盖面，大力发展特种水泥、水泥深加工，持续提升行业竞争力。橡胶产业，着力抓好丰源轮胎(二期)等项目建设，带动橡胶加工、炭黑、钢帘线等上下游产业发展。煤电产业，加快推进十电扩建、八一热电联产等项目建设，按照节能、清洁、环保要求，深化煤电合作运营。机械机床产业，发挥国家机床质检中心的标准引领作用，加快研发具有自主知识产权的新产品，培植壮大威达重工、三合机械等龙头企业，重点发展高端精密数控机床，提升装备制造业整体实力。玻璃产业，着力推进中国玻璃城、省玻璃质检中心建设，加快产业整合重组，增强高端产品研发生产能力，打造国内重要的玻璃深加工产贸基地。纺织服装产业，抓好青纺联等优势企业，做强产业龙头，引导中小企业分工协作、上下游配套，创立自主品牌，抢占高端市场。食品加工产业，依托农产品资源优势，发展农产品深加工，培育祥和、春藤等知名品牌，形成具有枣庄特色的食品品牌体系。

加快培育壮大新兴产业。编制新一轮战略性新兴产业发展规划，加强扶持引导，促进规模扩张。新医药产业，重点发展生物技术药、化学创新药、现代中成药和医疗器械，抓好华润三九、威智百科、康力医疗等项目，打造国内重要的中医药颗粒剂生产基地。新能源产业，以创建国家锂电池质检中心为带动，加速推进海特电子等项目，壮大锂电产业规模；抓好中广核风电等项目建设，创建太阳能应用示范城市。新材料产业，加快推进中材科技、东方光源等项目，支持企业开发特种高分子材料、纳米材料，力争年内达到百亿产业规模。新信息产业，抓住国家实施“互联网+”行动计划的机遇，加速信息技术与制造业跨界融合，推进通信网络基础工程建设，做大软件和服务外包基地等载体，培育电子商务、智能制造、云计算、物联网等新兴业态。节能环保产业，重点发展节能技术装备、污染防治药剂、废弃物综合利用技术，抓好天畅环保、中科生活垃圾发电等项目建设，培植绿色循环经济。

加快发展现代服务业。抓好布局优化、业态完善、产业融合，推动生产性服务业与生活性服务业协调发展。文化创意产业，制定产业功能区建设发展规划，推进台儿庄国家级文化产业园、石榴文化博览园、聚艺谷等文化创意园建设，打造文化产业交易平台和公共服务平台，培育一批专精新特文化领军企业。商贸物流产业，抓好鲁南铁路物流中心等项目，推进真爱商城等专业市场建设，支持企业建立电子商务网站、利用第三方平台开展网络营销，大力发展互联网物流。旅游休闲产业，抓好台儿庄古城、微山湖古镇等景区建设，提升冠世榴园、抱犊崮等景区管理水平，培育一批生态旅游特色镇、特色村，积极发展观光采摘、娱乐健身等新兴业态，建设环城游憩带，打造枣庄乡村旅游品牌。健康养生产业，抓好市社会福利(养老)服务中心、残疾人综合康复中心、银光养老中心等项目建设，鼓励社会资本创办老年康复中心、健康查体中心、老年公寓，构建高中低端互补的城乡养老服务网络，把“夕阳事业”做成“朝阳产业”。市场化办好节会活动，提高节会经济质量和效益。围绕完善生产要素交易市场、中介市场，积极培育研发设计、金融保险、产权交易、检验检测等生产性服务业，逐步形成业态全、消费足、市场旺的发展态势。

加快发展现代农业。围绕国家现代农业示范区建设，加快转变农业发展方式，提高农业规模化、产业化、标准化、品牌化、高效化水平。区域化布局特色产业，重点培育10个现代农业示范镇、20个特色农产品标准园和10个特色农业示范基地。积极引进国内外知名食品加工企业，拉长产业链条，带动农民增收致富。继续实施粮食高产创建活动，培育一批省、市级示范方，推动镇村整建制高产创建。加快培植新型农业经营主体，大力发展家庭农场，启动实施新型职业农民培育工程。抓好“一村一品”示范村镇建设，新培育区域农产品品牌3个以上，新认证“三品一标”农产品30个以上。统一内销与出口食品标准，健全质量安全追溯体系，创建出口农产品质量安全示范市。抓好生态农业与农村新能源示范县建设，开展永久基本农田划定工作，实施耕地质量提升规划和农业面源污染防治，加大土壤污染治理力度。加快农业机械升级换代和推广应用，减轻农民种地的劳动强度。推广节水灌溉技术，探索立体种养、生物共养循环生产模式。

加快实施创新驱动发展战略。充分发挥科技对经济社会发展的引领支撑作用，更多依靠人才资源、技术进步推动经济增长。突出企业创新主体地位，积极创建高层次创新平台。发挥市产业技术协同创新中心作用，深化与高等院校、科研院所的合作，围绕重点产业实施创新专项计划，突破一批关键技术，增强产业、企业、产品的核心竞争力。抓好科技企业孵化器建设，打造科技成果转化基地，助推新兴产业发展。加强中小企业公共服务平台建设，大力推进个转企、规下转规上、规上转股改、股改企业挂牌上市，出台鼓励大众创业、万众创新的扶持政策，让更多“创客”脱颖而出，促进民营经济蓬勃发展。实施英才集聚工程，突出产业发展、科技创新、教育医疗等重点领域，力争用五年左右的时间，引进培养100名创新创业领军人才，为转型发展提供强有力的智力支撑。

五、着力扩大对外开放

坚持引进来与走出去、扩大内需与争取外需相结合，大力优化营商环境，加快建立开放型经济新体系。

突出抓好招商引资。进一步挖掘培育差异化的比较优势，加快转变引资引智方式，增强对资本、人才、技术的吸引力。加强市场研判和产业对接，高标准筛选、策划、包装项目，紧盯京沪、江浙、港台和“一带一路”合作国家，务实开展精准招商、专业招商、主题招商。充分发挥企业的主体作用，推动以商招商、产业链招商，支持山能、中联、华电、联想等企业在枣庄增加投资。完善项目审批前置会商机制，强化对重大项目的要素保障，确保项目招得来、落得下、见效快。

突出抓好园区发展。按照配套集中、要素集约、产业集聚的要求，引导企业、项目到园区发展，打造产业培育的重要载体、转型发展的重要支撑和对外开放的重要窗口。建立项目跨园区落地利益协调机制，引导各个园区培育优势主导产业，形成特色鲜明、优势互补的园区发展格局。健全市场化、多元化的投入机制，吸引社会资本参与园区基础设施建设。完善项目考核退出机制，加快实施“腾笼换鸟”，提高园区土地节约集约利用水平。充分发挥枣庄高新区的引领作用，推动高新技术产业加快发展。支持滕州经济开发区创建国家级园区。

突出抓好外经外贸。设立国际贸易服务中心，扎实开展外贸企业跨境电子商务试点，重点引进一批外贸综合服务企业。积极组织企业参加境内外展会活动，做大做强机电机床、化工建材、纺织服装、农产品深加工等产品出口基地，大力开拓境外新兴市场。支持骨干优势企业走出去，到境外投资办企、建立原料基地、开展技术合作。抓住国际市场大宗商品价格走低的时机，适度增加关键设备、精密零部件和民生消费品等产品进口，促进外贸进出口协调增长。发挥好海峡两岸交流基地的作用，密切人员往来，扩大经贸合作成果。六、统筹城乡一体化发展

坚持以人为本、规划引领、产业支撑、生态优先、功能完善，加大以城带乡、城乡统筹的力度，着力提高新型城镇化质量。

推进规划建设管理一体化。实施全域城镇化战略，以中心城区为龙头，以区(市)城区为主体，以示范镇为节点，以农村新型社区和美丽乡村为支撑，构建功能明确、产城融合、协调发展的现代城镇体系。推进国民经济和社会发展规划、城市总体规划、土地利用总体规划等“多规合一”，加强各专项规划及市、区(市)规划之间的相互协同，强化规划的引领作用和刚性约束。拓宽市场化投融资渠道，引导社会资本通过股权融资、项目融资、特许经营等方式，参与城乡基础设施建设。加强重点城建项目市级统筹，促进重大基础设施合理布局、共建共享。坚持盘活存量与调控总量有机结合，引导建筑市场健康发展。严格落实属地管理责任，搭建统一高效的城市管理信息平台。启动智慧城市建设试点，让市民享受信息化的方便与快捷。

推进中心城区发展一体化。突出新城的龙头带动作用，建成启用市妇保院新院(一期)、文体中心，实施实验高中(三期)、职业中专(二期)和特殊教育学校迁建工程，启动体育运动学校建设，打通黄河东路、长江路，加快推进新城食品药品检验检测中心、图书大厦、金融大厦建设，高标准做好城市亮化工程，加速人气商气文气聚集。继续下大气力抓好老城改造提升，推进供排水、燃气、热力管网改造和主次干道、老旧小区、背街小巷、户外广告治理，完成市立医院门急诊大楼主体工程，加快十五中东校重建(二期)工程。坚持产业耦合、功能互补，推动薛城区、高新区错位发展，市中区、峄城区融合发展，提升中心城区综合承载能力。

推进基础设施建设一体化。注重城市基础设施向农村延伸，坚持地上与地下并重，完善城乡公共基础设施配套。完成北留线山亭至滕州段改建及6条国省道改造，开工建设枣庄高铁换乘枢纽、枣曹线桥梁改建及万年闸复线船闸工程，加快推进港航工程和新城至滕州快速公交项目，完成枣鱼高速东延、沂台线南延、枣济线改建前期工作，启动通用机场、郯薛线改建前期工作；新建改造农村公路300公里，逐步构建衔接顺畅的现代综合交通体系。全面开展地下管线普查，启动城市地下综合管廊和防洪防涝建设试点。建成餐厨废弃物处理设施，实现城市餐厨垃圾集中收集、无害化处理和资源化利用。加快城镇生活污水处理设施建设，示范镇生活污水处理率达到80%以上。实施“气化枣庄”工程，扩大城乡集中供应天然气范围。合理规划建设城市公共停车场、过街通道、应急避难场所等功能设施，促进地上地下空间同步开发、综合利用。

推进基本公共服务一体化。围绕促进人的城镇化，深化户籍管理制度改革，完善居住证和实际居住人口登记制度，推动农村转移人口市民化，加快城镇常住人口基本公共服务全覆盖。有序开展农村新型社区“村改居”，逐步纳入城镇化管理。积极培育城镇二三产业，促进农民就近就业、就地城镇化。推进美丽乡村建设，持续开展城乡环境综合整治，巩固提升城乡环卫一体化成果，加快创建国家卫生城市。深化“四德”工程建设，积极开展文明社区、文明家庭评选活动。推动城市优质教育、卫生、体育、养老资源向农村延伸，逐步实现城乡基本公共服务均等化。

推进生态建设一体化。围绕创建国家环保模范城市和省级生态市，铁腕治污、铁面执法，坚决打好节能减排和环境治理攻坚战。突出植树造绿，抓好荒山绿化、城乡绿色廊道建设等十大提升工程，巩固好国家森林城市创建成果；重点实施城区裸露土地、公园、小区和单位庭院等绿化工程，力争创建成为国家园林城市。突出治气治水，加大工业废气、城市扬尘、机动车尾气、超限超载治理和秸秆禁烧力度，淘汰黄标车3.2万辆，全面完成建成区燃煤锅炉替代改造任务；完善“治用保”水污染防治体系，加快重点治污项目建设，强化对排污企业的监管和执法。突出生态保障，严厉打击非法开采，抓好水土流失、破损山体、采矿塌陷地等生态脆弱区治理，增强生态自我修复能力。

七、大力发展民生和社会事业

新常态下经济发展面临换挡调速，群众更加期盼民生改善的“加速度”。各级政府务必把保障改善民生当成最大的事，千方百计保基本、补短板、兜底线，多些雪中送炭，多办惠民实事。

积极推动创业就业。引导全社会转变就业观念和创业方式，建设充满活力的创业型城市。实施更加积极的创业就业政策，广泛开展创业活动，全面激活各类创业主体。加强创业载体建设，争取新建9处市级以上创业示范园。组建枣庄创业大学，支持本地高校建设大学生创业园。积极开发公益性就业岗位，帮助特困家庭、“双零”家庭和高校毕业生就业。年内新增城镇就业4.2万人，农村劳动力转移就业5.6万人。

健全社会保障体系。进一步加大对社保领域的财政投入，逐步提高覆盖面和保障水平。城乡居民基本养老保险基础养老金由每人每月75元提高到85元。城乡居民基本医疗保险财政补助标准由每人每年320元提高到380元，实现省内联网医院即时结算；基本公共卫生服务经费补助标准由人均35元提高到40元。继续提高企业退休人员养老金标准。全面落实困境儿童基本生活保障制度，每人每月发放生活费不低于300元 金庸小说。新建保障性安居工程1万套以上，改造农村危房1500户。全面实施临时救助制度，构建综合性社会救助平台，规范发展慈善机构和慈善捐助。扎实开展精准扶贫，减少贫困人口15%以上。

统筹发展社会事业。加强薄弱学校改造，新建改建标准化校舍10万平方米、农村中小学食堂100个。高质量推进中小学素质教育，稳步实施中考制度改革。积极推行县级公立医院综合改革，推进分级医疗和双向转诊。实施城乡医院对口帮扶，选派500名医生到镇街卫生院、社区卫生服务中心开展诊疗服务。综合治理出生人口性别比，提高出生人口素质。办好第三届全国智力运动会。在200个村居建设体育健身广场。推进住宅小区物业服务星级评定。新建社区老年人日间照料中心30处、农村幸福院60处、市级妇女儿童家园14个。实施212个村电网改造和8万农民饮水安全工程。扩大公交、燃气、供电、供水等领域“一卡通”支付覆盖面。

加大文化惠民力度。制定优秀传统文化传承发展规划，深入挖掘墨子、鲁班、奚仲等传统文化和近现代中兴工业文化，大力弘扬铁道游击队等红色文化，保护传承柳琴戏、伏里土陶等民俗文化，培养一批有情怀、有担当的文化人才，创作一批富有时代特色和地域风格的文艺精品。扎实推进可移动文物普查和“乡村记忆”工程，加强文化遗产保护。抓好省级基层综合文化中心试点建设，完成100个农家书屋数字化改造。规范提升社区艺术团、庄户剧团，丰富城乡文化生活。

加强社会治理创新。把维护社会稳定作为第一责任，加大矛盾纠纷排查调处力度，深化“平安枣庄”建设。推行网格化社会管理，建设10个“四社联动”示范社区。强化属地管理，扎实开展安全隐患整治，提高应急处置能力，促进安全发展。深入实施“食安枣庄”行动，实现城区大型超市农产品监管检测全覆盖，着力治理“餐桌污染”。抓好农村市场产品质量监管，严厉打击制假售假行为。加强双拥共建。科学编制“十三五”规划。推动民族宗教、外事侨务、知识产权、档案史志、妇女儿童、红十字、残疾人、老龄、人防、地震、气象等各项事业全面发展。

八、进一步加强政府自身建设

当前，改革步入深水区、发展转入新常态，各级政府必须以敢于担当的精神和自我革新的勇气，践诺笃行搞服务，身体力行转作风，依法守规办事情，加快推进政府治理能力现代化。

坚持为民勤政。持之以恒反对“四风”，继续下大气力整改突出问题，巩固党的群众路线教育实践活动成果。完善直接联系和服务群众机制，使领导干部公开接访、主动下访成为常态，及时回应和解决群众的合理诉求。深入开展群众满意度测评、社情民意调查，积极推行电视问政、网络问政，提高市长热线、市长信箱、政风行风热线的办理质量。倡树立说立行、马上就办的工作作风，对定下来的事情，不出虚招、不放空炮，确保抓实办成。

坚持依法行政。树立法治思维，善用法治方式，建设法治政府。制定实施法治政府建设规划纲要，让行政权力在法律框架内运行。完善政府法律顾问工作机制，建立重大决策终身责任追究制度及责任倒查机制，做好行政复议应诉工作。整合执法主体，相对集中行政执法权，健全行政裁量权基准制度。坚持以公开为常态、不公开为例外，完善政府信息发布制度，全面推进政务公开和各领域办事公开，提高政府工作透明度。认真执行人大及其常委会决议、决定，积极办理人大代表建议和政协提案，主动听取民主党派、工商联、无党派人士和人民团体的意见，自觉接受媒体的舆论监督和群众的社会监督。

坚持科学施政。把发展为先、民生为本、稳定为要作为遵循，向群众问计、用事实说话，不断提升思想境界、工作标准和发展标杆，多做打基础、利长远、惠民生的实事好事。注重调查研究，使各项决策切合实际、便于实施。加强对市场经济理论的学习，积极运用辩证的思维和发展的眼光想问题、办事情。完善市政府工作规则，出台重大行政决策程序规定，严格执行公众参与、专家论证、风险评估、合法性审查、集体讨论决定的决策程序。坚持重质效、可持续原则，完善“差异定位、特色发展、分类考核”机制，提升区域经济发展水平。

坚持廉洁从政。各级政府及工作人员要严明纪律、严守规矩，本色做人、干净做事。严格落实党风廉政建设责任制，加强教育引导，筑牢思想防线。巩固“两集中两到位”成果，健全部门内控机制，推进网上审批系统、电子监察系统和公共资源交易平台建设。针对权力、资金、资源集中的部门和岗位，突出工程招投标、政府投融资、国有土地出让等重点领域，加强行政监察、审计监督和财政监管，坚决查处各类违法案件。深入贯彻落实中央“八项规定”精神，严肃财经纪律，严格责任追究，形成正风肃纪、刚性约束的正效应。

各位代表!事业凝聚人心，实干成就梦想。让我们紧密团结在以习近平同志为总书记的党中央周围，在市委的坚强领导下，解放思想、振奋精神，携手并肩、砥砺前行，共同谱写枣庄转型发展、科学发展、赶超发展的新篇章!
